# Spontaneous Rupture of a Choledochal Cyst

**DOI:** 10.5334/jbsr.2717

**Published:** 2022-01-17

**Authors:** Eduardo Bandeira, Rita Carneiro, Eugénia Soares

**Affiliations:** 1 Centro Hospitalar Lisboa Ocidental, PT; 2 Centro Hospitalar e Universitário de Lisboa Central, PT

**Keywords:** bile duct cyst, spontaneous rupture, biliary peritonitis, pediatric radiology

## Abstract

**Teaching point:** Spontaneous perforation with biliary peritonitis is a rare complication of bile duct cysts which should be considered in a patient presenting with acute abdomen, ascites, and dilated biliary tree on imaging.

## Case

A previously healthy 8-month-old female patient, born after an uneventful pregnancy, was admitted with a seven-day history of vomiting, acholic stools, abdominal distension, and mild fever. She was medicated with cefuroxime for a presumed urinary tract infection five days before attending our institution.

At physical examination, she had marked abdominal distension and signs of ascites, without a palpable mass. Laboratory investigation showed leukocytosis, total and conjugated hyperbilirubinemia, and elevated gamma glutamyl transferase.

**Figure 1:**
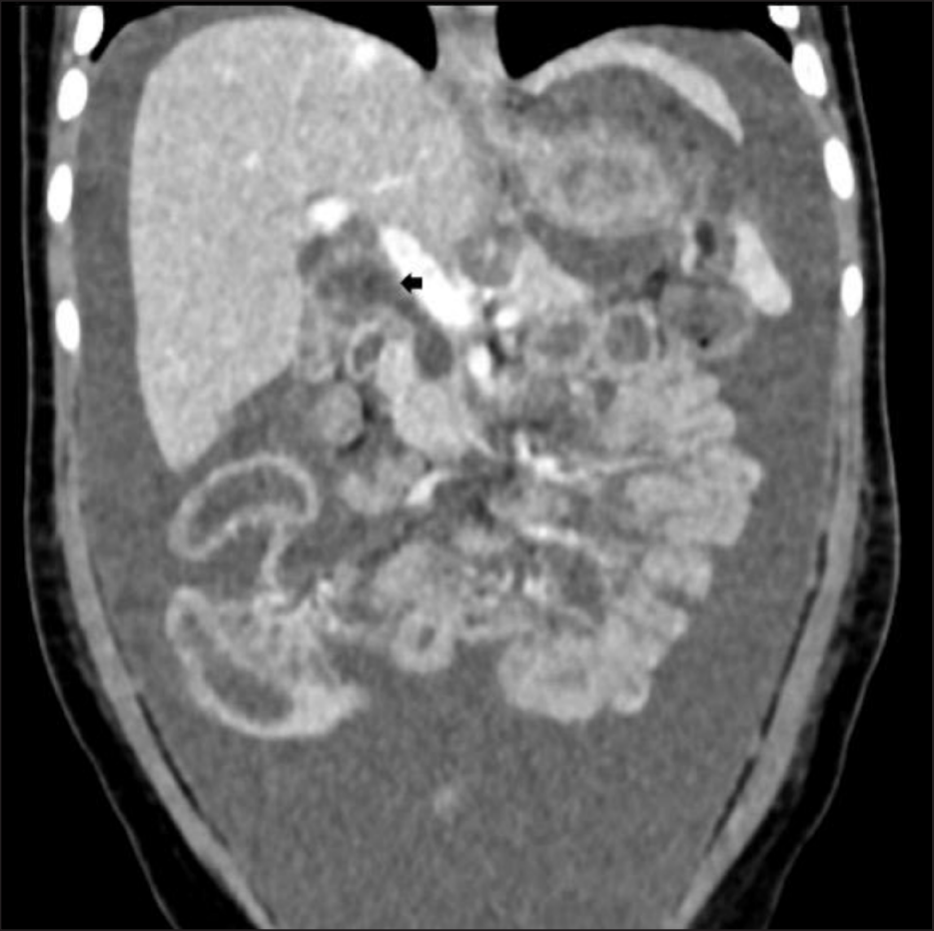


**Figure 2:**
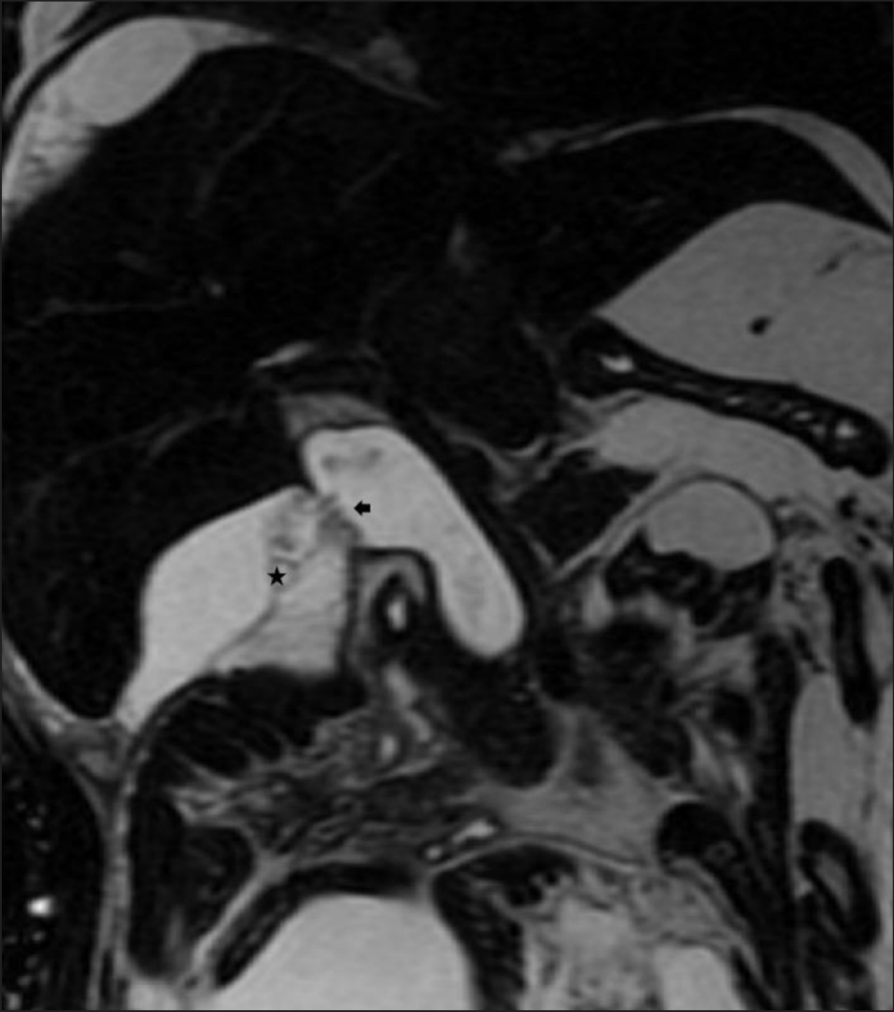


Ultrasound imaging showed substantial free ascites and a collapsed gallbladder. The extra-hepatic bile duct was obscured by abdominal gas. The intra-hepatic bile ducts and the liver had normal ultrasound characteristics. Coronal reformatted computed tomography (CT) (***Figure 1***) showed a dilated fusiform extrahepatic bile duct (arrow), which suggested the presence of a Todani 1 bile duct cyst. A ruptured choledochal cyst was suspected and confirmed on T2-weighted magnetic resonance cholangiopancreatography (MRCP) in the same plane as in ***Figure 1*** (***Figure 2***), which showed a > 5mm wall defect in the choledochal cyst wall (arrow) next to a peri-choledochal collection (star).

## Comments

Bile duct cysts are congenital dilatations of the intra-hepatic and/or extra-hepatic biliary tree. Todani devided these cysts in five groups. Type I cysts, the most common, consist in saccular or fusiform dilatations of the common bile duct. Known complications of bile duct cysts include lithiasis, cholangitis, and malignancy. Spontaneous perforation with biliary peritonitis is a rare complication of bile duct cysts, described in less than 2% of cases, usually in children [1]. The diagnosis can be suspected in a patient with acute abdomen, ascites, and dilated biliary tree on imaging. MRCP is the non-invasive method of choice to characterize bile duct cysts. As in our case, it can even show the perforation site in the cyst wall.
